# High risk of ischaemic stroke amongst patients with hereditary haemorrhagic telangiectasia

**DOI:** 10.1111/ene.16128

**Published:** 2023-11-13

**Authors:** Mikkel Seremet Kofoed, Pernille M. Tørring, Alex Alban Christensen, Bibi Lange, Anette Drøhse Kjeldsen, Troels Halfeld Nielsen

**Affiliations:** ^1^ Department of Neurosurgery, Odense University Hospital, Clinical Institute University of Southern Denmark Odense Denmark; ^2^ BRIDGE (Brain Research—Inter Disciplinary Guided Excellence) University of Southern Denmark Odense Denmark; ^3^ Department of Ear, Nose and Throat Surgery, Odense University Hospital, Clinical Institute University of Southern Denmark Odense Denmark; ^4^ University of Southern Denmark (SDU) Odense Denmark; ^5^ OPEN Region of Southern Denmark Odense Denmark; ^6^ Department of Clinical Genetics Odense University Hospital Odense Denmark; ^7^ VASCERN HHT Reference Center Odense Universitetshospital, Syddansk Universitet Odense Denmark; ^8^ Department of Neurology, Odense University Hospital, Clinical Institute University of Southern Denmark Odense Denmark

**Keywords:** cerebral abscess, hereditary haemorrhagic telangiectasia, ischaemic cerebral stroke, pulmonary arteriovenous malformations

## Abstract

**Background and purpose:**

Hereditary haemorrhagic telangiectasia (HHT) is a genetic disease with fragile blood vessels and vascular malformations, potentially causing neurological manifestations, including stroke and cerebral abscesses. The study aimed to investigate neurological manifestations in the Danish HHT database, focusing on pulmonary arteriovenous malformations (PAVMs) as a risk factor for cerebral events.

**Methods:**

Retrospective analysis of the Danish HHT database was conducted, cross‐referencing neurological outcomes with the Danish Apoplexy Register for accuracy. Patients were stratified by HHT type. Primary outcomes included ischaemic stroke, transient ischaemic attack and cerebral haemorrhage. Secondary outcomes comprised age, age at HHT diagnosis, age at cerebral ischaemic event, and PAVM and cerebral arteriovenous malformation status.

**Results:**

Six hundred and sixty‐four HHT patients were included. PAVM was diagnosed in 54% of patients, with higher prevalence in HHT type 1 (70%) compared to HHT type 2 (34%) and juvenile polyposis HHT (66%). Ischaemic stroke or transient ischaemic attack occurred in 12.5%, with a higher risk associated with macroscopic PAVM. Logistic regression showed a nearly 10 times increased risk of ischaemic stroke with macroscopic PAVM. Cerebral abscesses occurred in 3.2% of patients, all with macroscopic PAVM. Incomplete PAVM closure increased cerebral abscess risk.

**Conclusion:**

This study provides valuable insights into the prevalence of neurological manifestations and vascular events in HHT patients. The presence of PAVM was associated with an increased risk of ischaemic stroke, highlighting the importance of early screening and intervention. The findings emphasize the need for comprehensive management strategies targeting both vascular and neurological complications in HHT patients, especially regarding secondary stroke prevention.

## BACKGROUND

Hereditary haemorrhagic telangiectasia (HHT), also known as Osler−Weber−Rendu disease, is an autosomal dominantly inherited genetic disease in which patients develop fragile blood vessels, with telangiectatic lesions in the skin, oral and nasal mucosa as well as the gastrointestinal tract. HHT has a reported prevalence in Denmark of 1:6500 [1].

Besides cosmetic symptoms from the telangiectasia [2], HHT patients frequently suffer from problematic and recurrent bleeding, most often nosebleeds, and anaemia is a common challenge. HHT may also cause vascular malformations in internal organs, known as arteriovenous malformations (AVMs), which can occur in the liver, lungs, spinal cord (rare) and brain. Previous studies have shown an increased prevalence of neurological morbidity in patients with HHT [3, 4].

The aim of this study was to investigate and clarify the prevalence of neurological manifestations, stroke, cerebral abscesses and vascular manifestations in the brain amongst patients in the Danish HHT database, to determine the role of pulmonary AVM (PAVM) as a risk factor for cerebral events and to investigate groups of patients with HHT at high risk of neurological manifestations.

### Hereditary haemorrhagic telangiectasia

Hereditary haemorrhagic telangiectasia (HHT) is a clinical diagnosis based on the Curaçao criteria [5], which include spontaneous recurrent epistaxis, telangiectasias (of the lips, oral cavity, fingers and nose), visceral lesions (gastrointestinal, lungs, liver, brain and spinal tract) and a family history of HHT in a first‐degree relative diagnosed using these same criteria. Patients who fulfil three or more out of the four criteria are classified as having definite HHT, whereas those who fulfil two of the four criteria are categorized as having possible or suspected HHT [5] (see Table 1). The clinical HHT diagnosis can be verified by genetic analysis in most cases [6].

**TABLE 1:** Curaçao criteria, diagnostic criteria for HHT [3].

The HHT diagnosis is	
Definite	If three criteria are present or genetical analysis with known HHT pathogenic variant
Possible or suspected	If two criteria are present
Unlikely	If fewer than two criteria are present
Criteria	
Epistaxis	Spontaneous, recurrent nose bleeds
2 Telangiectases	Multiple and at characteristic sites: Lips Oral cavity Fingers Nose
3 Visceral lesions	Such as Gastrointestinal telangiectasia (with or without bleeding) Pulmonary AVM Hepatic AVM Cerebral AVM Spinal AVM
4 Family history	A first‐degree relative with HHT according to these criteria

Abbreviations: AVM, arteriovenous malformation; HHT, hereditary haemorrhagic telangiectasia.

Genetically, HHT is a heterogeneous disorder, caused by pathogenic variants in at least three genes; ENG, ACVRL1 and SMAD4. HHT type 1 (HHT1) is caused by pathogenic variants in the gene for endoglin (ENG) [7, 8, 9, 10] and accounts for 47% of Danish HHT patients [6]. HHT type 2 (HHT2) patients have pathogenic variants in ACVRL1 [10, 11, 12, 13] and constitute 41% of Danish HHT cases [6]. A mixed syndrome of HHT and juvenile polyposis (JP‐HHT) is caused by pathogenic variants of SMAD4 [10, 11, 12] and makes up 5% of Danish HHT patients [2]. Approximately 10% of Danish HHT patients meeting clinical criteria have no identifiable pathogenic variants in these genes [6].

Patients with HHT are at risk of neurological manifestations as a direct consequence of haemorrhage from cerebral AVMs (CAVMs) or indirectly from abscesses and paradoxical emboli due to PAVMs [14].

### Arteriovenous malformations (AVMs)

Arteriovenous malformations (AVMs) are clusters of abnormal vessels, commonly with a central nidus, receiving blood of high pressure from arteries, shunting it directly to the low‐resistance veins and bypassing the intervening capillaries, thus dilating the thinner walled veins with risk of potential rupture, the steal phenomenon and/or symptoms from compression of surrounding tissue [15].

### Pulmonary arteriovenous malformation (PAVM)

Pulmonary AVM (PAVM) has a reported prevalence in patients with HHT of 23%–65% [16, 17, 18, 19]. The risk of rupture from PAVM is low, 1%–8% [20], but an increased rupture risk has been observed during pregnancy [21].

Shunting of blood from the arterial to the venous system in PAVMs bypasses the functional tissue of the lungs, thus enabling unoxygenated and unfiltered blood to flow to the left atrium creating an associated risk of stroke or cerebral abscess and lower oxygen saturation [22]. Treatment of PAVM has shown a significant decrease of these risks [23, 24, 25]. Treatment of PAVM has been demonstrated to be effective and safe [25].The Danish HHT centre began endovascular treatment of PAVM in 1996, as reported by White et al. [24].

### Arteriovenous malformation of the brain (CAVM)

Cerebral AVMs are known to affect approximately 10% of patients with HHT. Patients with HHT1 are known to have a higher incidence of CAVM (13.4%) compared to patients with HHT2 (2.4%) and JP‐HHT [26, 27].

Previous data suggested a lower risk of bleeding in HHT‐associated CAVM compared to sporadic CAVMs, 0.41%–1.73% versus 2.0%–2.7% yearly risk of rupture [28, 29, 30]. However, on comparing the rebleeding risk of ruptured HHT‐associated CAVMs with the rebleeding risk of sporadic CAVMs, it is found to be significantly higher at 10.07% versus 4.8%, respectively [4]. The lower risk of first‐time bleeding in HHT‐associated CAVM might partly be explained by the increased number of scans on asymptomatic HHT patients compared to the general population, giving rise to identification of more CAVMs that potentially will never rupture.

The data from Brinjikji et al. [26] suggest that 20% of all patients with HHT‐related CAVM will suffer from cerebral haemorrhage and 50% have symptoms from the CAVM, including headache, seizure and/or focal neurological deficits.

## METHODS

Data included in the study were collected retrospectively from the prospectively maintained Danish HHT database. The STROBE guidelines [31] have been adhered to in designing our study and in the drafting of the paper.

Neurological outcomes of all patients in the database have been cross‐referenced with the Danish Apoplexy Register (DAP database) [32] to ensure optimal quality of registered neurological outcomes. The DAP database has collected information on ischaemic stroke and transient ischaemic attack (TIA), amongst other outcomes, of Danish patients admitted to hospitals across all regions of Denmark since 2003.

Patients were stratified according to HHT type. Primary outcome was one of the following cerebral events: ischaemic stroke, transient ischemic attack (TIA) or cerebral haemorrhage. Secondary outcomes were age at HHT diagnosis and age at registered cerebral ischaemic event, together with PAVM and CAVM status.

Pulmonary AVM was defined as either macroscopic or microscopic. Macroscopic PAVM was defined as visible PAVM on a computed tomography (CT) scan of the thorax. Microscopic PAVM was defined as a positive screening with transthoracic contrast echocardiography [33], with visible bubbles after four heart beats but with no visible PAVM on CT scans of the thorax.

Cerebral AVM was defined as either nidal type or pial arteriovenous fistulas. All CAVMs were diagnosed using brain magnetic resonance imaging. Aneurysms, cavernomas, capillary malformations and deep venous anomalies were not classified as CAVM. Ischaemic stroke was defined as registered ischaemic cerebral stroke event or TIA. Two patients have documented cerebral events without symptoms (silent brain infarction) and were registered as ischaemic stroke.

### Danish HHT database

The Danish HHT centre has been situated at Odense University Hospital since 1996 and all HHT patients seen at the centre have been included in the national research database. In 2015 data were uploaded in a RedCap database and since then all patients included in the database have received written information and been given informed consent. Data regarding date of birth, age at diagnosis, HHT type, results of screening for PAVM, CAVM and hepatic AVM are included. Furthermore, data regarding age of onset of epistaxis, results regarding date of neurological events, cerebral ischaemia, bleeding and abscess are also included in the Danish HHT database.

The Danish HHT database is located at Odense University Hospital, in RedCap GDPR (General Data Protection Regulation) certified servers. It has been approved by the Danish Data Protection Agency (ID 15/10194) and the Region of Southern Denmark's regional secretariat of law (ID 22/22675).

### Statistical analysis

Statistical analyses were done using STATA 17. All analyses have been approved by a biostatistician.

Dichotomous categorical data were analysed for their degree of independence with the chi‐squared test. Continuous data were investigated for normal distribution with the Shapiro–Wilk test, and one‐way ANOVA was used to test significant levels of difference between the five groups. If data were not normally distributed the Kruskal−Wallis test was used to test significance.

Multivariate analyses were done using logistic regression and, in the case of non‐binary ordered data, ordered logistic regression adjusting for age and gender was used.

All primary analyses were pre‐planned and used to confirm existing hypotheses, rather than generating new ones.

## RESULTS

### Patients

A total of 664 patients were registered in the Danish HHT database, as of 1 February 2023. All patients in the Danish HHT database were diagnosed with HHT according to the Curaçao criteria, and all patients were included in the study. HHT1 accounted for 326 patients, 273 had HHT2 and 31 patients had JP‐HHT. In 34 patients, HHT was diagnosed according to the Curaçao criteria but without genetic confirmation. Of these, no pathogenic variants could be identified by genetic analysis in 22 patients, and no genetic analysis was performed in 12 patients.

The mean ages across the groups were as follows: HHT1 (55 years), HHT2 (56 years) and JP‐HHT (42 years), with age ranges spanning from 6 to 99 years. The mean ages at the time of diagnosis were HHT1 (38 years), HHT2 (41 years) and JP‐HHT (35 years), ranging from 0 to 92 years. See Table 2.

**TABLE 2:** Patient demographics.

HHT type	1 (ENG)	2 (ACVRL1)	JP‐HHT (SMAD4)	Clinical HHT	Not genetically tested	Total	*p* value
Number	326	273	31	22	12	664	–
Age							
Mean	55	56	42	64	77	59	<0.001
Median	56	57	42	62	78	59	–
Mean at HHT diagnosis	38	41	35	46	44	41	0.015
Cerebral event, mean age	53	65	46	32	62	53	0.413
Sex							0.632
Men	160	126	17	8	6	317	–
Women	166	147	14	14	6	347	–
CAVM							
Confirmed	16	1	0	2	0	**19**	**<0.001**
Ruled out	72	43	7	5	0	127	–
Treated	11	2	0	1	0	14	–
Aneurysm	2	0	2	1	0	5	–
Other vascular malformation	7	3	0	0	0	10	–
PAVM							**<0.001**
Macro	158	35	13	8	3	217	–
Micro	44	44	6	1	1	96	–
Total	202	79	19	9	4	**313**	–
Ruled out	87	153	10	12	1	263	–
PAVM treated	133	28	8	8	3	180	–
Embolized	125	25	8	7	3	168	–
Surgical management	5	3	0	1	0	9	–
Embolized and surgical	3	0	0	0	0	3	–
Cerebral abscess							
Confirmed	17	2	0	2	0	**21**	**0.009**
Cerebral event							
Stroke or TIA	60	14	2	4	3	**83**	**<0.001**
Haemorrhage	9	1	0	0	0	10	0.077
No known event	256	259	29	18	9	571	–

*Note*: Statistically significant differences between HHT types are marked in bold with their *p* value.

Abbreviations: CAVM, cerebral arteriovenous malformation; HHT, hereditary haemorrhagic telangiectasia; JP‐HHT, juvenile polyposis hereditary haemorrhagic telangiectasia; PAVM, pulmonary arteriovenous malformation; TIA, transient ischemic attack.

### Pulmonary AVM

A total of 576 patients were screened for PAVM. PAVM was diagnosed in 313 patients (55%), with 217 macroscopic and 96 microscopic PAVM. Stratified according to HHT type, 202 (70%) of examined HHT1 patients (158 macroscopic/44 microscopic), 79 (34%) of examined HHT2 patients (35 macroscopic/44 microscopic) and 19 (66%) of examined JP‐HHT patients (13 macroscopic/six microscopic) had PAVM. Clinical HHT and unknown HHT mutation status in conjunction account for 13 patients (50%) with PAVM (11 macroscopic/two microscopic). See Table 2.

Logistic regression showed a significant difference between the groups, with a 4.5 times increased risk of PAVM in HHT1 compared to HHT2 (odds ratio [OR] 4.49, *p* < 0.001, 95% confidence interval [CI] 3.10–6.51), and a 3.7 times increased risk in JP‐HHT compared to HHT2 (OR 3.68, *p* = 0.002, 95% CI 1.63–8.29).

Logistic regression showed a significant difference between genders, with women having 1.45 times higher risk of PAVM (OR 1.45, *p* = 0.026, 95% CI 1.04–2.02). Neither HHT type nor gender changed their level of significance with multivariate logistic regression.

### Stroke

Of all patients with HHT, 83 (12.5%) met the primary outcome of TIA or stroke. Within the last 15 years 39 strokes have been reported, indicating a 3.9/1000 patient‐year risk of cerebral ischaemic stroke or TIA. Mean age for stroke or TIA event was 53 years, range 0–53, throughout the groups; see Table 2. The chi‐squared test of independence was performed to assess the relationship between stroke and PAVM, as shown in Table 3. There was a significant relationship between the two variables with chi‐squared value 26.44, *p* < 0.001.

**TABLE 3:** Distribution of ischaemic stroke event stratified according to pulmonary arteriovenous malformation (PAVM) status.

Stroke	No	Yes	Total
No PAVM	329	22	351
PAVM	252	61	313
Total	581	83	664

*Note*: Chi‐squared value 26.44. *p* < 0.001.

Dichotomized into macroscopic PAVM or not, the PAVM and stroke relationship still showed a significant relationship between the two variables, with a chi‐squared value of 52.18, *p* < 0.001. See Table 4.

**TABLE 4:** Distribution of ischaemic stroke event stratified according to known macroscopic pulmonary arteriovenous malformation (PAVM) status.

Stroke	No	Yes	Total
No PAVM	329	22	351
Microscopic PAVM	91	5	96
Macroscopic PAVM	161	56	217
Total	581	83	664

*Note*: Distribution of PAVM classified by stroke event. Chi‐squared value 52.18. *p* < 0.001.

Logistic regression investigating the effect of patient PAVM status on the risk of ischaemic stroke, adjusting for age and gender, showed a significant 9.77 times increased risk of ischaemic stroke for macroscopic PAVM versus no PAVM (OR 9.77, *p* < 0.001, 95% CI 4.72–20.19). There was no significant correlation between microscopic PAVM and the risk of having ischaemic stroke (OR 1.74, *p* = 0.335, 95% CI 0.57–5.34). Patients who had not undergone screening for PAVM had a non‐significantly increased risk of ischaemic stroke (OR 2.25, *p* = 0.09, 95% CI 0.88–5.73).

Gender was not associated with the risk of ischaemic stroke in either the multivariate logistic regression above or by a chi‐square test of independence, *p* = 0.930. Age was significantly associated with the risk of ischaemic cerebral stroke, *p* < 0.001 (95% CI 1.03–1.06).

### Cerebral abscess

A total of 21 patients (3.2%) in the Danish HHT database were previously diagnosed with cerebral abscesses. Of those 21 patients, all were found to have PAVM; in fact, all of these patients had macroscopic, as opposed to microscopic, PAVMs; see Table 5.

**TABLE 5:** Distribution of cerebral abscesses classified by pulmonary arteriovenous malformation (PAVM) status (no known PAVM, macroscopic and microscopic PAVM).

Cerebral abscess	No	Yes	Total
No PAVM	351	0	351
Macroscopic PAVM	196	21	217
Microscopic PAVM	96	0	96
Total	643	21	664

In 21 HHT patients diagnosed with cerebral abscess, data on the degree of closure of the PAVM were found in 18 patients; see Table 6. Of these 18, five patients were diagnosed with cerebral abscess after initial treatment of their PAVM; four of these patients had small PAVMs left for follow‐up, and one had a major PAVM awaiting further treatment.

**TABLE 6:** Known events of cerebral abscesses stratified according to degree of pulmonary arteriovenous malformation (PAVM) closure.

Degree of treatment	Abscess before first treatment	Abscess after PAVM treatment	Unknown	Total
Complete closure of PAVM	8	0	0	8
Small residual PAVM	1	4	0	5
Large residual PAVM	4	1	0	5
Unknown treatment status	0	0	3	3
Total	13	5	3	21

*Note*: Chi‐squared value 10.024. *p* = 0.007.

A chi‐squared test of independence, investigating the association between the risk of cerebral abscess after treatment of PAVM and the degree of closure, showed a statistically significant association with chi‐squared value 10.024, *p* = 0.007.

### Cerebral AVM

As of 1 February 2023, 146 patients have received a brain magnetic resonance imaging for the purpose of identification of CAVMs. Nineteen patients (13%) have CAVM, of whom 16 have HHT1, one has HHT2, none has JP‐HHT and two have clinical HHT; see Table 2.

Ten patients (6.8% of those screened and 1.5% of total patients in the database) have had a cerebral haemorrhage. Seven of these patients had been diagnosed with CAVM, two patients with aneurysms and one patient had a haemorrhagic stroke at only 2 days of age; subsequent investigations did not find evidence of vascular malformations in this patient.

The difference in risk of CAVM based on our data suggest that HHT1 has a 14 times increased risk of CAVM compared to HHT2 (OR 14.04, *p* = 0.011, 95% CI 1.85–106.5).

Statistical analysis did not show a significant association between the risk of CAVM and gender.

## DISCUSSION

The Danish HHT database excels due to its highly systematic approach. The HHT centre is located at Odense University Hospital and covers all five regions of Denmark. With 664 patients, it is thought to capture approximately 82% of the expected total number of HHT patients in Denmark (1:6500) [1], and with verified pathological HHT associated mutations in 95% of the patients it is expected that our data are highly representative of HHT.

### Pulmonary AVM

In Denmark, PAVMs began being treated by an endovascular approach in 1996–1998. Before this, treatment of PAVM meant surgery, and in some cases lobectomy, and was thus reserved for highly symptomatic PAVMs. The introduction of endovascular treatment increased efficacy and safety of treatment and a systematic screening for PAVM in all HHT patients ensued. In the Danish HHT centre, all new patients and all patients seen in follow‐up are offered and recommended to screen for PAVM.

Our data showed a prevalence of macroscopic PAVM of 32% in accordance with data from previous reports [16, 17, 18, 19]. The data also showed an increased risk of macroscopic PAVM in HHT1 (48%) and JP‐HHT (42%) compared to HHT2 (13%).

### Stroke

The Danish background population has a 2.6/1000 patient‐year incidence of cerebral stroke or TIA, with a mean age of 72 and a median age of 74 years [34]. The prevalence of ischaemic stroke in the Danish HHT database was 83/664, with 39 reported strokes within the last 15 years, giving rise to a 3.9/1000 patient‐year risk of cerebral ischaemic stroke or TIA. Mean age of ischaemic stroke or TIA throughout the HHT types is 55 years of age.

By separating the groups into their PAVM status it was found that patients with macroscopic PAVM had a 9.52/1000 patient‐year incidence of ischaemic cerebral stroke or TIA. Patients with microscopic PAVM had an incidence of 2.08/1000 and patients with negative screening of PAVM had a 1.52/1000 patient‐year incidence of ischaemic cerebral stroke or TIA.

An incidence of 9.52/1000 patient‐years of ischaemic cerebral stroke or TIA is an approximately 3.5 times higher incidence compared to the Danish background population (2.6/1000 patient‐years). Whilst comparing rates of incidence from a small group to the Danish background population has several problems, it does show a surprisingly high incidence, and does confirm the previously reported high incidence of ischaemic stroke in HHT [4] as being limited to HHT patients with macroscopic PAVM.

Several studies have tried to investigate the effect of the severity of PAVMs on the risk of cerebral ischaemic events, with mixed results [4]. Until now, there has not been a threshold to determine with certainty when a PAVM is clinically relevant regarding the risk of cerebral ischaemic events. The data presented, with a total of 313 patients with PAVM, suggest that microscopic PAVM, defined as a positive screening with transthoracic contrast echocardiography but no visible PAVM on CT scan of the thorax, should not be considered clinically significant regarding the risk of cerebral ischaemic events.

Previous studies have shown that treatment of PAVM lowers the associated risk of ischaemic stroke and cerebral abscesses, with a high rate of success [23, 25], but patients with HHT frequently have multiple PAVMs, and it is unclear whether treating the largest of several PAVMs lowers the risk of stroke significantly and whether prophylactic platelet inhibitors can be discontinued upon complete closure of a PAVM after prior ischaemic stroke. It has not been possible to investigate these questions in the current study, but further investigation into this matter is certainly warranted.

### Cerebral abscess

It was found that 100% (21/21) of patients in our cohort who had a cerebral abscess all had macroscopic PAVMs. Data on date and degree of treatment were found for 18 of these, of which 13 patients presented with a cerebral abscess before the diagnosis and treatment of PAVM. All five patients who presented with a cerebral abscess after their initial PAVM diagnosis and treatment were patients with incomplete closure of their PAVM (four small PAVMs left for follow‐up and one larger PAVM awaiting further treatment). There are no patients in our database who have been diagnosed with a cerebral abscess after complete closure of their PAVM, despite these patients no longer being prescribed prophylactic antibiotic treatment for dental work.

These findings support the current practice of prophylactic antibiotic treatment for patients with PAVM prior to dental visits and support that the need for prophylactic antibiotics ceases in cases of complete closure. It also reinforces the importance of achieving complete closure together with routine rescreening to investigate possible revascularization or development of new PAVM.

A correlation has been shown between macroscopic PAVM and the risk of both ischaemic stroke and cerebral abscesses. No significant evidence was found to suggest that microscopic PAVM increases the risk of either cerebral abscesses or ischaemic stroke.

Currently all HHT patients over 12 years of age who have not yet received screening for PAVM, or have been diagnosed with a pulmonary shunt, including microscopic PAVM, are recommended to take prophylactic antibiotics prior to situations with increased risk of bacteraemia (oral surgery, dental work etc.). The absence of patients with microscopic PAVM in the cerebral abscess group raises the question: do HHT patients with microscopic PAVM need prophylactic antibiotic treatment?

Our data could be a product of the protection against cerebral abscesses from the prophylactic antibiotics that are recommended, but it is known that compliance in this regard is far from 100% and the complete absence of patients with microscopic PAVM with cerebral abscesses suggests that prophylactic antibiotics may not be needed; however, more data are needed before any firm conclusions can be drawn.

Our data do help emphasize the risk to patients with macroscopic PAVM, even in the setting of partial treatment of their PAVM and prophylactic antibiotic treatment, and rapid complete closure of macroscopic PAVM must be a high priority.

### Cerebral AVM

Currently the Danish HHT centre does not systematically screen asymptomatic patients with HHT for CAVMs. Hence, the data from the Danish HHT database do not represent the presupposed CAVM prevalence. Currently as of 1 June 2022, 19 of 664 patients have diagnosed CAVM (2.6%), with 14 of 17 seen with HHT1, giving it a grouped prevalence of 4.3%.

In 2011 the first international guidelines describing the clinical diagnosis and management of HHT patients recommended systematic screening of HHT patients with the goal of seeking out CAVM. These recommendations were affirmed in 2020 with the second international guidelines [35]. The European Reference Network published a position statement in 2020 stating that systematic screening of HHT patients for the goal of seeking out CAVM is not recommended [36]. Currently no studies have investigated whether the positive effect of systematic screening for CAVM outweighs the negative, and so current recommendation is based on consensus statements. The difference in recommendation is rooted in the often‐unpredictable nature of CAVM in HHT patients with a relatively low yearly risk of rupture (0.43%) [30], together with the non‐negligible treatment‐related risk of CAVM (mortality and morbidity rate of 0.3% and 2.2% respectively [15]).

In our cohort, 1.5% of the patients have had a cerebral haemorrhage. Whether this percentage warrants screening of all HHT patients is a question it is currently not possible to answer.

## CONCLUSION

Our main goal was to investigate the prevalence of neurological manifestations of HHT in the Danish HHT database and to correlate cerebral events with the presence of CAVM and/or PAVM.

It was found that macroscopic PAVM, visible on CT of the thorax, significantly increases the risk of cerebral ischaemic stroke and is highly associated with the risk of cerebral abscesses in patients with HHT.

With this study, the aim was also to validate current standard procedures of screening and management of PAVM. Our data suggest that microscopic PAVM does not significantly increase the risk of cerebral events of either ischaemic cerebral stroke or cerebral abscess.

Our data contribute to and support the importance of screening and treating patients with HHT for PAVM. Further investigation is needed to clarify the often problematic issue of antiplatelet treatment in patients with HHT and stroke, and specifically when it can be avoided or discontinued.

## AUTHOR CONTRIBUTIONS

Mikkel Seremet Kofoed: Conceptualization; investigation; funding acquisition; writing—original draft; methodology; validation; visualization; software; formal analysis; project administration; data curation. Pernille M. Tørring: Writing—review and editing; supervision; data curation; formal analysis. Alex Alban Christensen: Writing—review and editing; validation; formal analysis; supervision. Bibi Lange: Resources; supervision; writing—review and editing. Anette Drøhse Kjeldsen: Data curation; resources; supervision; project administration; writing—review and editing; investigation; conceptualization; methodology; validation; formal analysis; visualization. Troels Halfeld Nielsen: Conceptualization; investigation; methodology; validation; visualization; writing—review and editing; resources; supervision; project administration.

## FUNDING INFORMATION

The corresponding author has received research support for salary in his position as a PhD student from the Region of Southern Denmark, the University of Southern Denmark and from a patient donation.

## CONFLICT OF INTEREST STATEMENT

The authors declare no conflict of interests.

## ETHICS STATEMENT

The Danish HHT database is located at Odense University Hospital, in RedCap GDPR (General Data Protection Regulation) certified servers. It is approved by the Danish Data Protection Agency (ID 15/10194) and the Region of Southern Denmark's regional secretariat of law (ID 22/22675).

## Data Availability

The data that support the findings of this study are available on request from the corresponding author. The data are not publicly available due to privacy or ethical restrictions.
